# Penile Skin Bridges after Circumcision

**Published:** 2015-09-01

**Authors:** Georgios Kampouroglou, Konstantinos Nikas

**Affiliations:** Department of Pediatric Surgery, Agia Sophia Children’s Hospital, Athens, Greece

**Dear Sir,**

Penile skin bridges are adhesion between penile shaft skin and the glans penis after circumcision.[1] They may tether the circumcised penis during erections, causing deformity and occasionally pain.[2] Skin bridges constitute healed surgical wounds and require division for correction.[1] 

We herein present a healthy 13-year-old adolescent, who complaint of tightening and tugging of his penis during erections. He was circumcised at the age of three year. Clinical examination revealed three skin bridges at 3, 11 and 12 o’clock position, with a width of about 2-3 mm each. These acted as bridles during erections (Fig. 1). The bridges were surgically excised under general anesthesia using electrocautery, with no bleeding. There was no recurrence three months after surgery.

**Figure:**
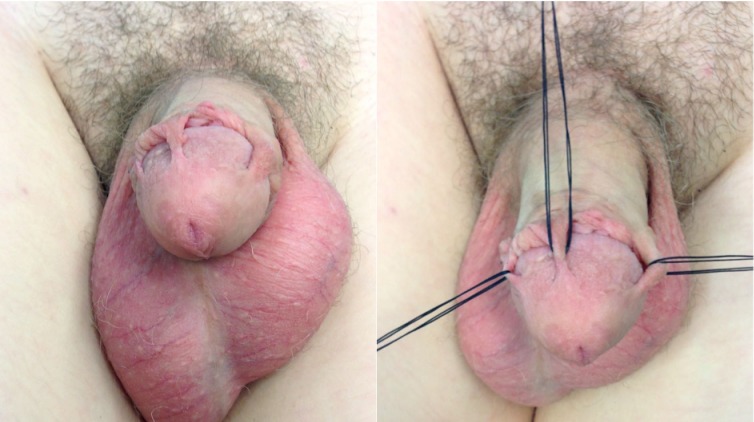
Figure 1: Penile skin bridges.

The etiology of this condition remains unknown. Several possible factors have been proposed, such as injury to the glans at the time of circumcision or incomplete separation of the inner preputial skin.[2,3] During healing, the shaft skin can adhere to the glans and in some cases the adhesions separate partially at the corona to form bridges.[4,5] In order to prevent the formation of penile skin bridges, careful suturing and good dressing at the time of circumcision should be done. Small bridges are narrow and avascular. These can be divided with the use of a silver nitrate stick. On the other hand, the thicker more vascular ones require the use of electrocautery as done in index case.

## Footnotes

**Source of Support:** Nil

**Conflict of Interest:** None declared

